# Process evaluation of a complex intervention in augmentative and alternative communication care in Germany: a mixed methods study

**DOI:** 10.1186/s12913-025-12452-y

**Published:** 2025-03-13

**Authors:** Sarah Anna Katharina Uthoff, Anna Zinkevich, Jens Boenisch, Stefanie Kalén Sachse, Tobias Bernasconi, Lena Ansmann

**Affiliations:** 1 Department of Health Services Research, Faculty of Medicine and Health Sciences, Carl Von Ossietzky University of Oldenburg, Ammerlaender Heerstrasse 140, Oldenburg, 26129 Germany; 2 Chair of Medical Sociology, Institute of Medical Sociology, Health Services Research and Rehabilitation Science (IMVR), Faculty of Medicine, University of Cologne, Eupener Str. 129, Cologne, 50933 Germany; 3 Department of Special Education and Rehabilitation, University of Cologne, Habsburgerring 1, Cologne, 50674 Germany; 4 Department of Special Education and Rehabilitation, University of Cologne, Klosterstr. 79b, Cologne, 50931 Germany

**Keywords:** Formative evaluation, AAC, Health services research, Qualitative research, Quantitative research, Focus group interview, Longitudinal intervention study

## Abstract

**Background:**

In Germany, clear care pathways for people without natural speech who require augmentative and alternative communication (AAC) are currently lacking. Therefore, AAC is often not sustainably implemented in everyday life. For this reason, a complex intervention was developed that supplements existing AAC consultation with additional AAC training, AAC therapy, and case management. This article presents the results of the process evaluation of the complex intervention. It examines (1) how caregivers and AAC consultants rate the intervention (2), which contextual factors influence its implementation, and (3) the acceptance, use competence, and use of the new AAC system.

**Methods:**

The process evaluation used a mixed methods design. Quantitative data were collected with a longitudinal survey of caregivers of AAC users in the intervention and comparison groups at three time points (T0: after AAC consultation; T1: 4 weeks after AAC system receipt; T2: 4 months after AAC system receipt). Semi-structured focus group interviews were conducted with caregivers of AAC users and with AAC consultants. The quantitative data were analysed descriptively and with nonparametric mean value comparisons. The qualitative results were analysed using structured qualitative content analysis.

**Results:**

The evaluation and presentation of results were based on the Medical Research Council process evaluation guidance by Moore et al. The intervention elements were rated positively. AAC training and therapy enabled the participants to use the AAC system effectively in different contexts. Case management provided support, particularly in the event of problems in the care process. However, the results also show the heterogeneity of the intervention, as it depended on various contextual factors. Overall, acceptance, use competence, and use of the AAC system were rated higher in the intervention group than in the comparison group.

**Conclusions:**

The process evaluation illustrates various contextual factors that can influence the implementation of the AAC intervention. The results highlight the potential of the intervention to improve AAC care and establish a sustainable use of AAC systems in everyday life. In addition, the process evaluation provides indications of how AAC interventions can be adapted for successful implementation.

**Trial registration:**

Grant number 01NVF17019.

**Supplementary Information:**

The online version contains supplementary material available at 10.1186/s12913-025-12452-y.

##  Introduction

Augmentative and alternative communication (AAC) encompasses a range of strategies designed to support individuals whose ability to communicate verbally is limited or impaired due to congenital or acquired disabilities. The natural speech skills of people who rely on AAC can be severely impaired (e.g., in the case of complex disabilities, in some cases of infantile cerebral palsy or global aphasia after stroke) or increasingly deteriorate as a result of neuro-degenerative processes (e.g., in the case of amyotrophic lateral sclerosis, multiple sclerosis or Parkinson’s disease) [1, 2]. Therefore, the group of people who rely on AAC is very heterogeneous in terms of age, underlying types of disabilities or medical conditions, and ranges from young children with complex multiple disabilities to people of older age [1, 2]. AAC is often used additionally when usual speech therapy interventions are not possible or sufficient due to the complexity of the disability. AAC strategies include the use of unaided AAC systems (e.g., gestures) and aided AAC systems (e.g., symbol cards, electronic devices with voice output) [1, 3–5]. The care needs of people with AAC often require holistic, interdisciplinary, and multisectoral care due to multiple complex health and social care needs [1, 6–8].

For Germany, reliable data on the prevalence of people who rely on AAC are lacking. A nationwide survey of German schools with a special focus on physical and motor development by Boenisch [9] showed that the number of children with limited verbal abilities at these specific schools is on average 20%. Data from the United Kingdom show that approximately 0.5% of the United Kingdom population has speech, language or communication needs and therefore has a need for AAC [2]. Beukelman & Light [1] estimated that approximately 5 million people in the United States of America and approximately 97 million people worldwide have complex communication needs and could benefit from AAC.

Every person with limited verbal abilities in Germany is authorised to receive an aided AAC system funded by statutory health insurance (SGB V § 32–33) [10]. However, there are still some potential care problems, such as the fact that AAC care is not clearly regulated and is characterised by unclear responsibilities. Furthermore, there is a risk of inappropriate provision of AAC systems, a lack of quality criteria and standards, a lack of follow-up care, and regional differences in care which can lead to frequent lack of use of AAC in everyday life [6, 9, 11–17].

In Germany, if an AAC system is applied to a health insurance company, AAC consultation from a medical equipment supplier is required [18]. Advice from third parties such as independent AAC counselling centres can be obtained by insured persons on their own initiative [15]. There are more than 100 AAC counselling centres in Germany that offer independent AAC consultations. However, many of the counselling centres are affiliated with schools and are primarily accessible to pupils with AAC needs. As AAC care via AAC counselling centres is not prescribed or regulated by law, they are often organised heterogeneously and offer different services [17, 19]. In comparison to AAC counselling centres, medical equipment suppliers do not provide independent consultation and therefore there is a risk of provision with AAC systems that is not needs-based [6, 12]. There are also occasional interruptions in the care process because the requested AAC systems are rejected by health insurance companies, and statements and objections must be submitted [6, 7]. Complex intervention studies on the effectiveness of improvements in AAC care, especially with a (quasi) experimental design are rare. Accordingly, there is a need for large-scale intervention studies related to improvements in AAC care [20–23].

### Complex intervention

Due to these potential problems in AAC care in Germany, a complex intervention, was developed, implemented, and evaluated within the project “New Service-Delivery Model for Augmentative and Alternative Communication (AAC) Devices and Intervention”. An intervention can be defined as complex if it possesses certain characteristics, such as involving multiple components, targeting a wide range of behaviours, requiring significant expertise and skills from those delivering or receiving it, addressing multiple groups, settings, or levels, or allowing a high degree of flexibility in its implementation or individual components [24].

As the project took place in cooperation with a health insurance company, only patients of this health insurance company could take part in the intervention. The intervention extends an existing selective contract, which includes independent AAC assessment and consultation at an AAC counselling centre [25].

**Fig. 1:**
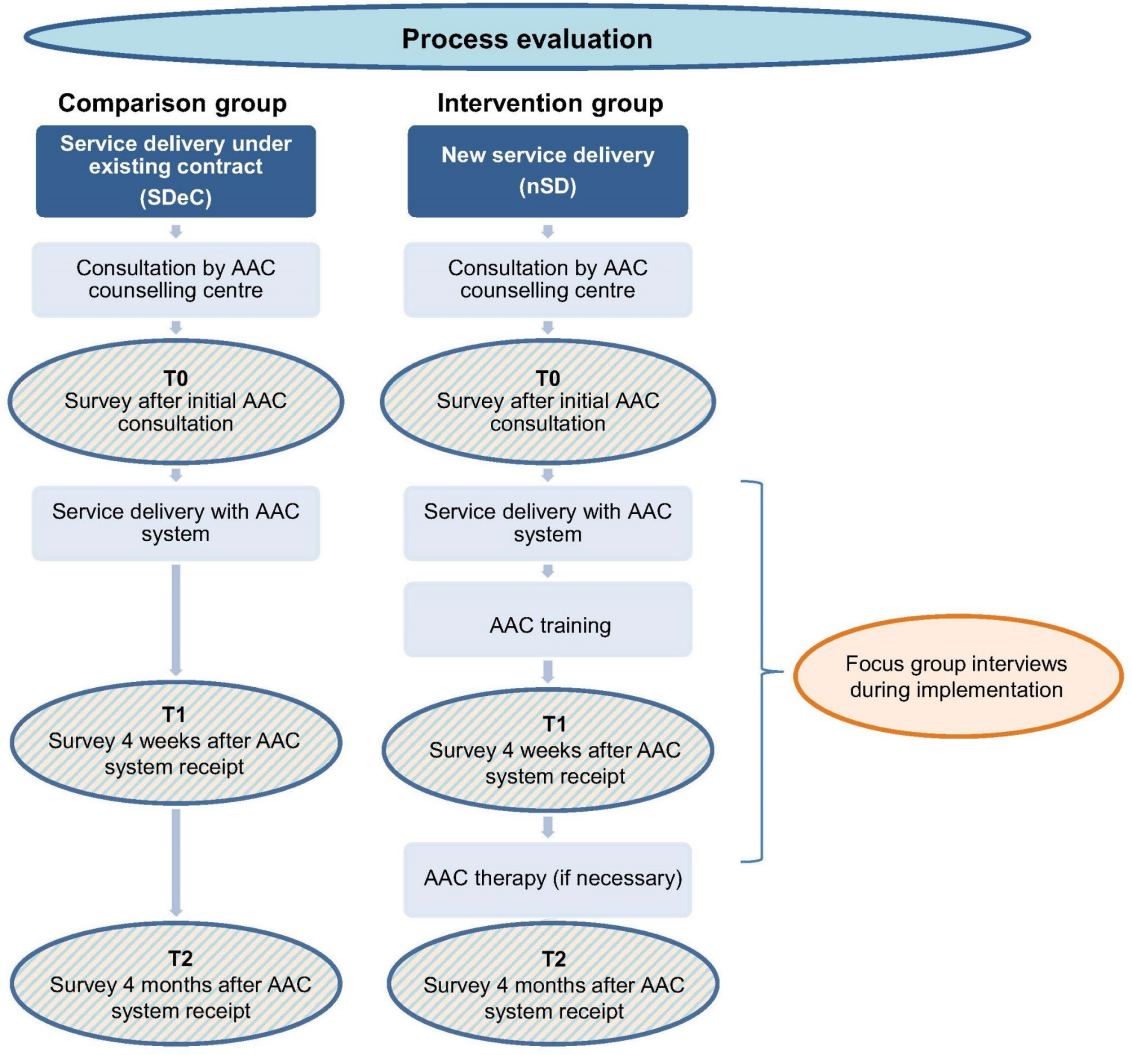
Study design of the process evaluation

Evaluations of the selective contract show that the measures of the selective contract are not sufficient to successfully implement AAC in the long term (Boenisch J, Schäfer K, Schellen J, Willke M: UK-Beratungen am FBZ-UK der Universität zu Köln. Evaluation, Analysen, Perspektiven. Unpublished evaluation report on AAC consultation at the FBZ-UK, Köln, unpublished). For this reason the intervention extends the provision of independent AAC assessment, and consultation with AAC training, (4 appointments) AAC therapy (20 appointments) and accompanying AAC specific case management (Fig. 1). This complex intervention was carried out by three participating AAC counselling centres [25]. AAC assessment and consultation was carried out within two appointments by AAC consultants, with a subsequent recommendation for a needs-based AAC system. Formal (e.g., teachers) and informal (e.g., parents) caregivers were invited to participate in the AAC assessment and consultation. AAC training aimed to teach AAC users and caregivers how to use the AAC system, e.g., by combining, adapting, and expanding vocabulary. The main aim of AAC therapy was to learn how to use the AAC system in various everyday situations with the stakeholders involved. The accompanying AAC specific case management coordinated, among other things, AAC care and communication between stakeholders. Through network analysis, case management identified all relevant stakeholders for participation in AAC care. In addition, case management was intended to prevent problems in the care process and to ensure the sustainability of the intervention [7].

**Fig. 2:**
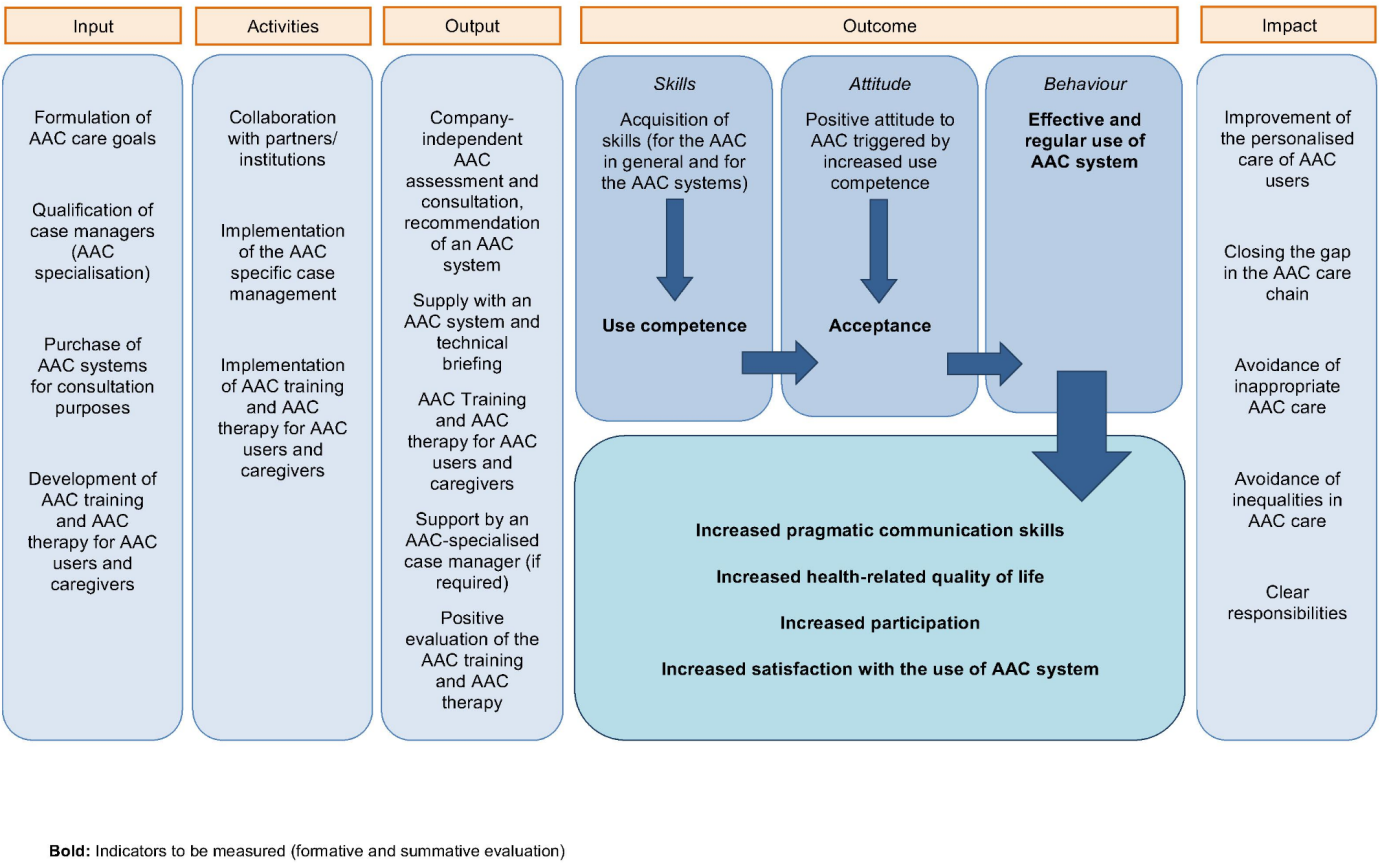
Logic model

The main goals of the intervention were to improve the pragmatic communication skills, health-related quality of life and participation of the AAC users as well as the satisfaction of the informal and formal caregivers with the AAC system. The overarching goal of the intervention was to improve continuity in AAC care and to close potential care gaps so that functional AAC system use and thereby significantly improved communication could be achieved [25]. Details about the project can be found in the study protocol [25]. The following logic model (Fig. 2) illustrates the suggested relationships between the intervention components and the indicators to be measured [25, 26].

### Aims and research questions

The study design included a process and outcome evaluation. The focus of the process evaluation was on the different components of the intervention, their implementation characteristics and influencing context factors [27]. Context factors are external elements like social, cultural, organisational, and policy conditions that influence the implementation and effectiveness of health interventions [24, 26]. This article presents the results of the process evaluation, which will be published before the results of the outcome evaluation to be able to first understand how the intervention was implemented and perceived. The aim was to investigate contextual factors that influence intervention implementation, to evaluate how the participants assess the components of the intervention, to assess intermediate outcomes as the use in everyday life, the use competence and the acceptance of the AAC system. The term “intermediate outcomes” refers to intermediate results or intermediate goals that are achieved on the way to the final goal of an intervention. These outcomes are indicators of whether an intervention is working in the desired direction and can be seen as milestones in the course of implementation [26]. The following questions were investigated:

1. How do the caregivers and AAC consultants rate the intervention?
2. Which context factors affect the implementation?
3. How is the use of the new AAC system of the AAC users in everyday life?
4. How competent are the AAC users and caregivers in using the AAC system?
5. How is the acceptance of the AAC users and their caregivers of the new AAC system?

## Methods

### Study design

The process evaluation is designed as a mixed methods study using qualitative focus group interviews and quantitative surveys to cover relevant perspectives of AAC users as well as their informal and formal caregivers [26, 28, 29]. A mixed methods approach was chosen to combine the strengths of quantitative and qualitative methods, allowing for a comprehensive understanding of the research questions. Quantitative data provides measurable insights into patterns and trends within and between the groups, while qualitative data captures the experiences and contextual factors of the participants who took part in the intervention. Details on the study design have been published in the study protocol [25].

Quantitative surveys of formal and informal caregivers of AAC users (T0: after initial AAC assessment and consultation; T1: 4 weeks after AAC system receipt [after AAC training]; T2: 4 months after AAC system receipt [after AAC therapy]) were conducted in the intervention group and in the comparison group (Fig. 1). Furthermore, focus group interviews with informal and formal caregivers who participated in the intervention as well as with AAC consultants of the three participating AAC counselling centres were conducted during implementation (Fig. 1). A convergent design is applied in the mixed methods study, whereby qualitative and quantitative data were collected concurrently in order to address the same research questions. Consequently, both types of data are given equal weight. We present the results of both methods in the results section and discuss these findings together in the discussion [30, 31]. The Reporting-Guideline ‘Good Reporting of A Mixed Methods Study (GRAMMS)’ according to O’Cathain et al. [32] was used to ensure the quality of the study.

### Sampling criteria and recruitment

The recruitment of AAC users in the intervention and comparison groups was conducted via the three participating AAC counselling centres. The inclusion criterion for AAC users in both groups was the presence of a congenital or acquired disability associated with no or limited natural speech. AAC users who had no natural speech due to hearing impairment were excluded from participation. AAC users with hearing impairment and multiple disabilities were an exception and were included. For the intervention group only AAC users insured by a specific health insurance company were eligible. AAC users insured by any other health insurance company participated in the comparison group. For every participating AAC user one informal caregiver and one formal caregiver were named and invited to participate in the quantitative longitudinal survey.

Homogeneous focus group interviews with AAC consultants as well as heterogeneous focus group interviews with formal and informal caregivers participating in the intervention were conducted. The recruitment of focus group participants during implementation was carried out using purposeful sampling [33] by caregiver status (formal or informal) and caregiver role (e.g., teachers, parents, personal assistants). In all surveys, the caregivers also acted as proxy respondents for the participating AAC users. Only caregivers and no AAC users were directly involved in the surveys reported in this article.

### Data collection

#### Quantitative data

The questionnaires were developed using cognitive pretesting (*n* = 16 pretest interviews with informal and formal caregivers of AAC users) [34]. Reminder procedures according to Dillman [35] were carried out at each survey time point with a postal reminder after 2 and 4 weeks. The process evaluation items were mostly self-developed and covered the following areas: caregivers’ assessment of intervention components and a variety of relevant context factors, such as the technology commitment of caregivers. Furthermore, sociodemographic information about AAC users and their caregivers was included. Acceptance, use competence, and use of the AAC system were assessed with a scale (subscale ‘acceptance of the caregivers’, 5 items; Cronbach’s α = 0.781) adapted from the study by Calculator [36, 37], which investigated the acceptance of AAC systems by individuals with Angelman syndrome. The caregivers’ technology commitment was measured using a validated scale (Cronbach’s α = 0.830) according to Neyer et al. [38]. Further survey results on the evaluation of collaboration with stakeholders and the caregiver burden have been published by Uthoff et al. [7] and Zinkevich et al. [39].

#### Qualitative data

The focus group interviews were conducted in the three AAC counselling centres participating in the project. All interviews were audio recorded and conducted using a semi-structured interview guideline [40–42] by two interviewers (SAKU, AZ) and one person for documentation. The two interviewers were research assistants on the project and were responsible for the evaluation. The person for documentation was a student assistant and also worked on the project. The interview guideline contained the following main questions:

- How was the intervention practiced?
- What are your experiences with the intervention?
- How would you rate the effects of the intervention?
- What adaptations need to be made to the intervention?

Both guidelines can be accessed in the supplement. Further results of the focus group interviews on the evaluation of collaboration with stakeholders and the caregiver burden have been published by Uthoff et al. [7] and Zinkevich et al. [39].

### Data analysis

The Medical Research Council (MRC) guidance on process evaluations of complex interventions by Moore et al. [26] was used at the data analysis stage with the aim of combining the quantitative and qualitative data in a structured way and presenting the evaluation aims. Furthermore, the MRC guidance was used to develop the category system for the evaluation of the focus group interviews and to synthesise the findings.

#### Quantitative data

The paper questionnaires were electronically scanned with the Electric Paper TeleForm software and a plausibility check was carried out. Subsequently, the data were analysed with the statistics programme IBM SPSS V.27. Quantitative data were analysed descriptively and by nonparametric mean comparison between the intervention and comparison groups using the Mann-Whitney U-test.

#### Qualitative data

The audio recordings were transcribed and subsequently analysed via structured qualitative content analysis according to Kuckartz [43]. For all the focus group interviews, the main categories were developed á priori on the basis of the main questions of the interview guideline and the MRC guidance: implementation, effects of the intervention implementation, outcomes, context factors, needs for adaption [26]. Afterwards some subcategories were formed deductively also according to the MRC guidance and some subcategories were developed inductively. The inductively formed subcategories involved analysing the data without predefined categories, allowing themes to emerge naturally. Two researchers (SAKU, AZ) coded all interviews using the software MAXQDA Analytics Pro 2020 (Release 20.4.0) independently and compared the results in a subsequent consensus process.

## Results

### Sample description

During the implementation of the intervention, a total of k = 7 focus group interviews (k = 3 homogeneous focus group interviews and k = 4 heterogeneous focus group interviews) were conducted with a total of *n* = 31 participants. On average, the focus group interviews lasted 92 min (73–111 min) [7]. See Table 1 for a detailed description of the sample.

**Table 1:** Sample description of the focus group participants [7]

Focus group participants’ characteristics (intervention group)	*n* (%)
**Total number of participants**	31 (100)
**Homogeneous focus groups** (**k = 3**, ***n*** **= 11)**	
AAC consultants	11 (35.5)
**Heterogeneous focus groups (k = 4**, ***n*** **= 20)**	
Informal caregivers (only parents)	8 (25.8)
Therapists	2 (6.5)
Educators and institutional employees	
Teachers	3 (9.7)
Employees of homes for persons with disabilities	2 (6.5)
Employees of sheltered workshops	1 (3.2)
Educators	3 (9.7)
Special needs teachers	1 (3.2)
**Sex**	
Female	25 (80.7)
Male	6 (19.4)
**Age groups**	
18–25 years	1 (3.2)
26–35 years	6 (19.4)
36–45 years	11 (35.5)
46–55 years	11 (35.5)
56–65 years	2 (6.5)

Due to rounding, percentages might not add up to exactly 100%

**Fig. 3:**
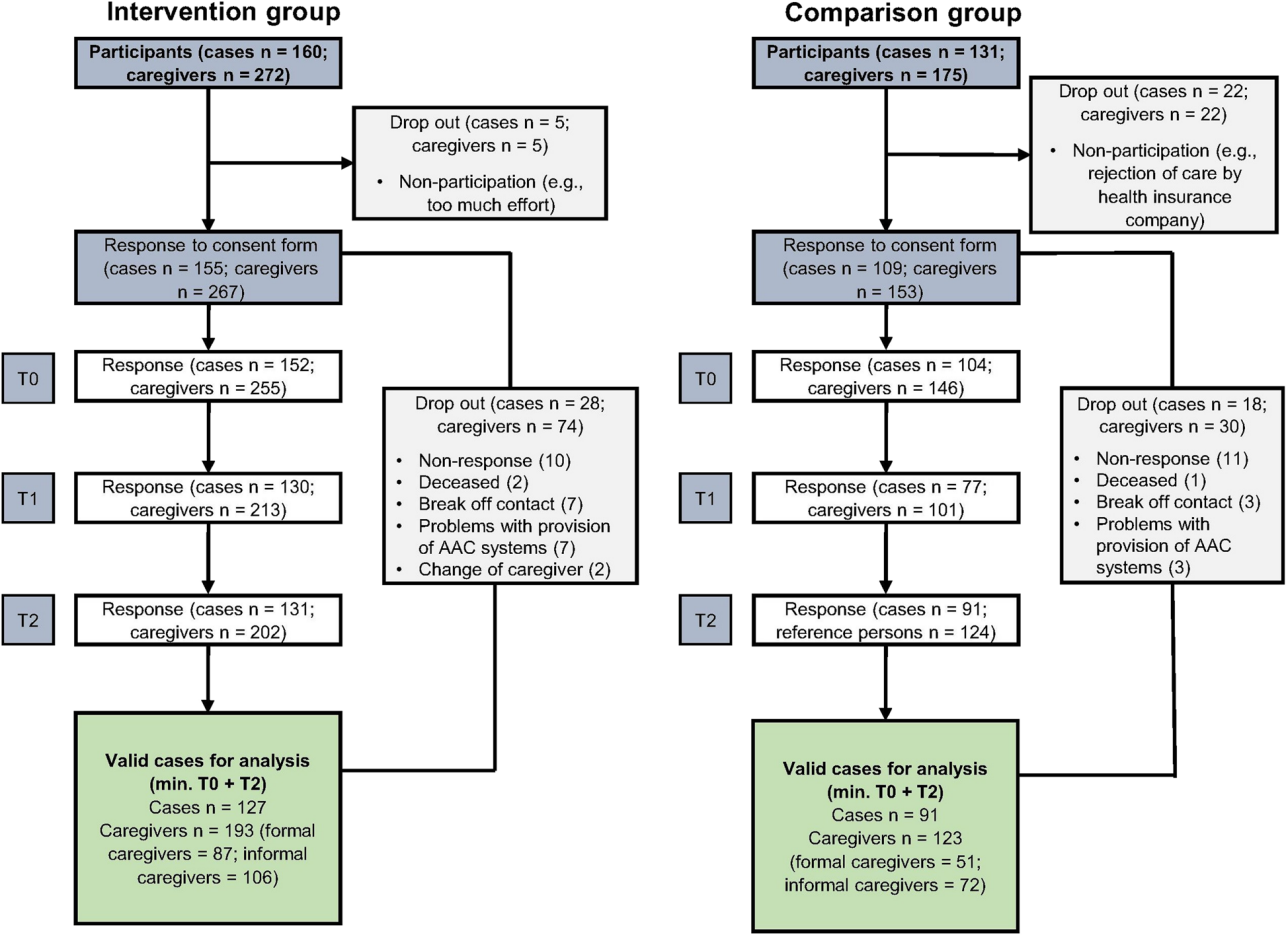
CONSORT flow charts of the intervention and comparison groups Note: The term “cases” refers to AAC users for whom questionnaires were completed by caregiversN

The original sample size and the drop outs of the intervention study can be seen in the CONSORT flow charts (Fig. 3). The final analysis sample comprised *n* = 127 cases/*n* = 193 caregivers in the intervention group and *n* = 91 cases/*n* = 123 caregivers in the comparison group. A detailed sample description of the participating AAC users and caregivers is provided in the Supplement. The power calculation for the outcome evaluation can be found in the study protocol [25].

### Evaluation results

**Fig. 4:**
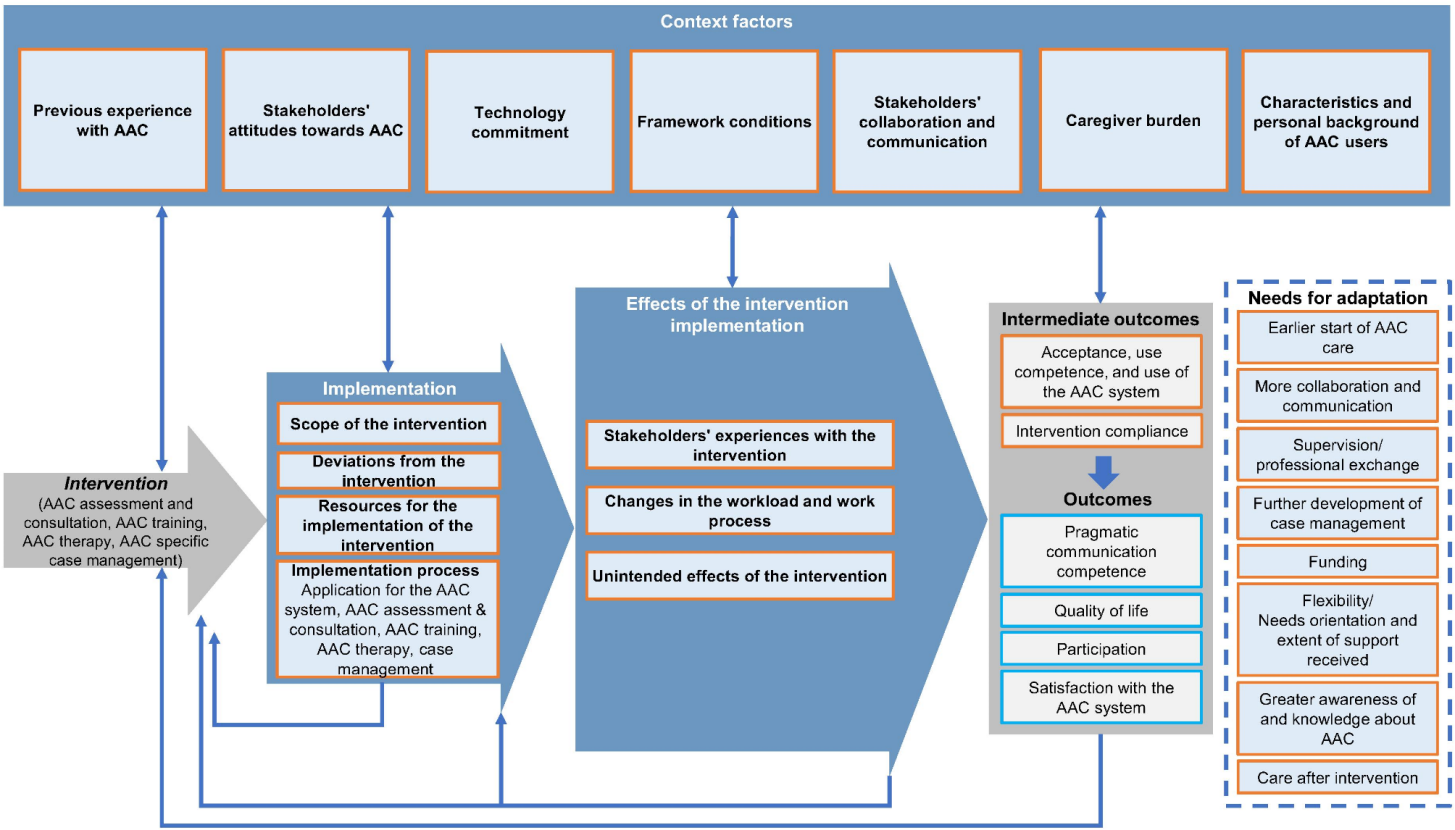
Synthesis of process and outcome evaluation based on Moore et al. [26] Note: Outcome evaluation endpoints are outlined in blue, and process evaluation endpoints are outlined in orange

The analysis is based on the MRC process evaluation guidance by Moore et al. [26]. Figure 4 synthesises the results based on the model and guides the presentation of the results. For the completeness of the evaluation results depicted in Fig. 4, indicators of the outcome evaluation (subcategory “outcomes” highlighted in blue) have been included, but will not be presented and discussed in this paper. However, we decided to include a set of intermediate outcomes (acceptance, use competence, use of the AAC system, and intervention compliance) in the process evaluation since they contribute to understanding the mechanisms of the intervention and are associated with the implementation of the intervention. The results summarised in Fig. 4 indicate how the intervention was implemented, the effects of the implementation, the context factors that influence the entire process, the influence of the intervention on the (intermediate) outcomes, and the identified needs for adaptation. The context factors result from inductively formed categories of the qualitative analysis (e.g., stakeholders’ attitudes towards AAC) as well as from factors explicitly assessed in the questionnaire (e.g., technology commitment). In the following, the mixed methods integration of quantitative and qualitative analyses is reflected in joint presentations of quantitative and qualitative results of the main categories of the model.

### Implementation

####  Scope of the intervention

As AAC therapy was only carried out on a needs-based basis, 12.6% of *n* = 127 cases included in the study did not receive AAC therapy. The majority of intervention participants (68.5%) received the intended amount of 20 therapy sessions. The intervention duration varied from 3 months to 27 months, and in most cases exceeded the planned duration of approximately 8 months (e.g., due to delays caused by the COVID-19 pandemic, hospitalisations, stays abroad, and scheduling difficulties). The survey periods (T0–T2) were longer than planned in both groups, which was mostly due to long waiting periods between AAC system application and delivery.

#### Deviations from the intervention

The results of the focus group interviews show that deviations from the intervention were caused e.g., by staff changes in the AAC counselling centres, by problems in scheduling the AAC training and therapy appointments, and by problems in the approval of AAC systems by health insurance companies. The following quote illustrates this result:

* “And of course it’s not refinanced at first*,* all this organisational work*,* the familiarisation*,* the understanding*,* the reading up*, so I was basically finished with a colleague once and then it started all over again and *name* had to work it all up somehow.” (B1CT1M,* AAC consultant*,* 26)*

#### Resources for the implementation of the intervention

The AAC consultants reported in the focus group interviews that the resources needed by the AAC counselling centres for the implementation of the intervention included among others case management training, familiarisation with specific intervention materials, adaptation of the overall care process to the intervention structures, and acquisition of AAC systems for consultation. The resources required for implementation are illustrated in the following quote:

*“(…) where I also think it’s important to have a large methodological… a big toolbox full of methods that you can fall back on in such cases*,* right? And that you have experience and can somehow manage it. And then*,* of course*,* you also have to be flexible.” (B1BT1M#1*,* AAC consultant*,* 74)*

#### Implementation process

The results of the focus group interviews revealed that even though the main elements of the intervention were standardised, the details of the intervention had to be adapted to the individual case. On the one hand, different stakeholders were involved in the individual AAC network. On the other hand, the individual cases differed according to the location of the AAC training and AAC therapy (at home, in the AAC counselling centre, or in institutions such as schools). The aim of AAC assessment and consultation was to test different AAC systems and to bring together all relevant stakeholders to recommend the most suitable AAC system for AAC users and their caregivers. The process of the application for the AAC system at the health insurance company was reported to be very heterogeneous, especially with regard to the time it took for the AAC system to be approved and delivered. It was not uncommon that statements and objections had to be submitted. In the AAC training sessions, the main goal was to ensure that the AAC users and their caregivers were able to use the AAC system. In this regard, many organisational and technical issues related to the AAC system have been addressed. In addition, AAC training included the development of AAC therapy goals. In AAC therapy, the use and transfer of the new AAC system into various everyday situations was practised to ensure its use. The case managers had the overall responsibility for the AAC care process and its coordination. In particular, case management was applied to more complex cases who needed particular support.

### Effects of the intervention implementation

#### Stakeholders’ experiences with the intervention

**Fig. 5:**
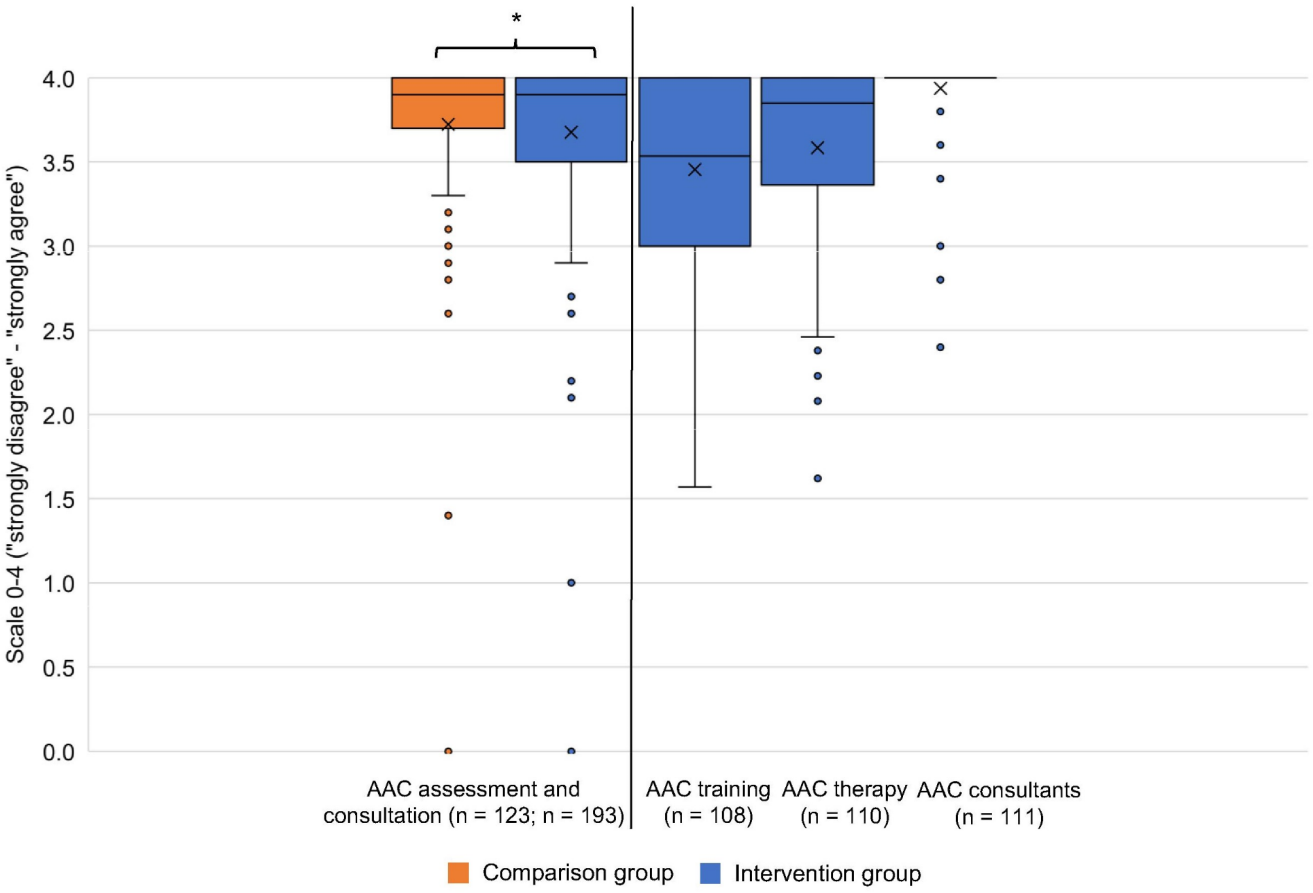
Assessment of the intervention elements and AAC consultants Note: Comparative values for the comparison group are only available for the AAC assessment and consultation construct. Higher values indicate a higher evaluation of the intervention components. Mann-Whitney U-Test: significant results are marked with * (*p* = 0.037)

The survey results showed that both groups rated the AAC assessment and consultation highly positively (Fig. 5). The mean value in the comparison group (values from 0 “strongly disagree” to 4 “strongly agree”; mean = 3.72) was slightly, but significantly higher than the value in the intervention group (mean = 3.68; Mann-Whitney U-test: *p* = 0.037). Since the AAC assessment and consultation should be performed equally in both groups, no difference in the evaluation was expected. The intervention group assessed AAC training (mean = 3.46), AAC therapy (mean = 3.59), and AAC consultants very positively (mean = 3.94).

In both groups, the caregivers were asked to evaluate case management. As previously published in a paper on the collaboration of stakeholders by Uthoff et al. [7], caregivers in the intervention group reported significantly more support from the AAC counselling centre in almost all aspects than did those in the comparison group. Caregivers in the intervention group also reported significantly fewer unmet support needs, e.g., defining goals for AAC care and networking the environment.

**Fig. 6:**
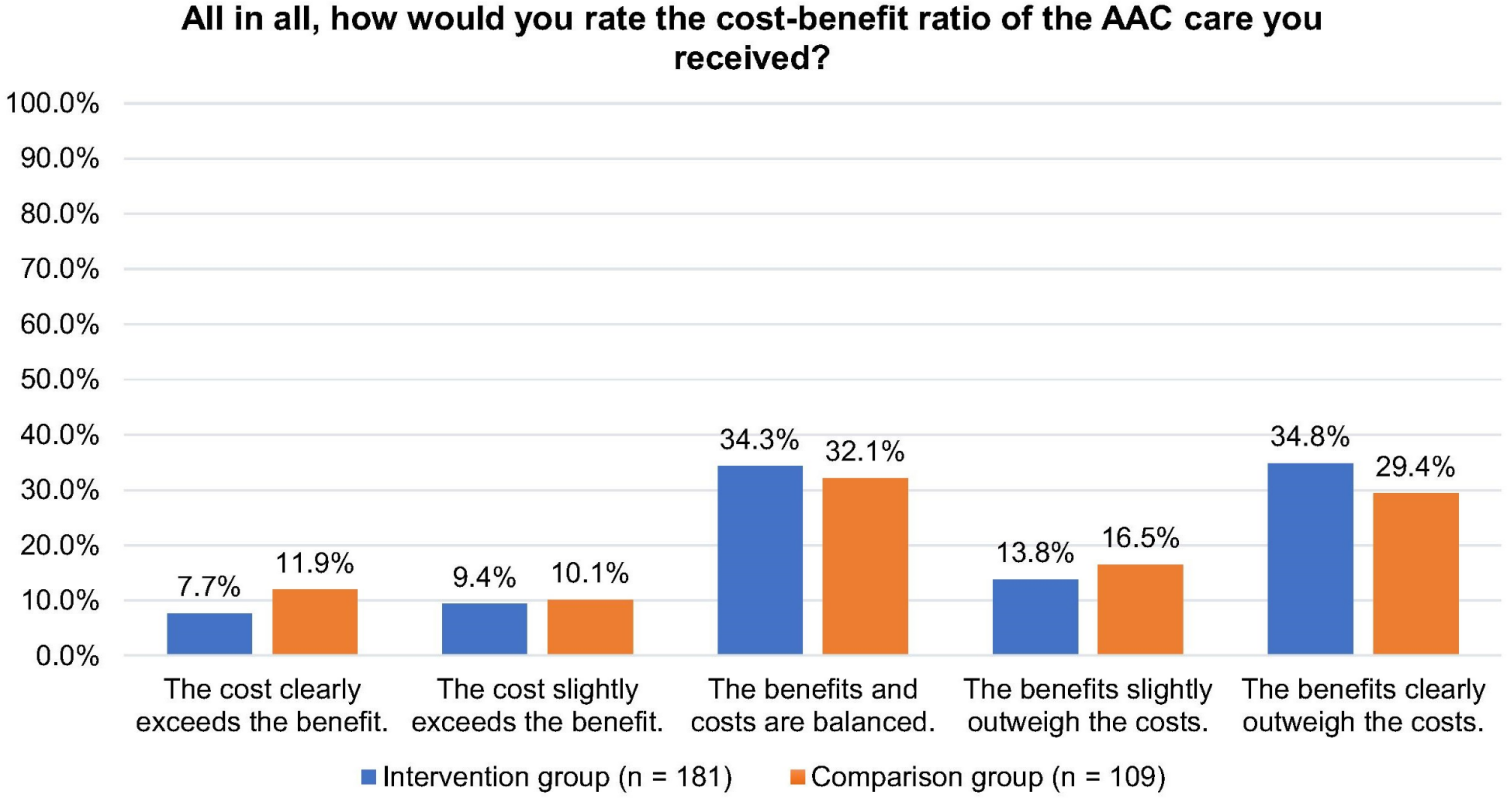
Overall assessment of the received AAC care

In order to better assess the overall evaluation of the intervention, the caregivers were asked in the questionnaire to rate the effort involved in participating in the intervention in comparison to the subjectively perceived benefits. The caregivers rated the benefit of the intervention higher than the effort needed to participate in the intervention, although one-third stated that the benefit and effort were balanced (Fig. 6).

The participants of the focus group interviews evaluated the elements of the intervention positively and helpful and identified the following impact factors on AAC care: needs-based choice of the AAC system, strengthened collaboration of all stakeholders, transfer of learned communication skills into everyday life, motivation and increase in the use of the AAC system, as well as the support received from AAC counselling centres. Problems in the implementation arose due to the lack of individual motivation and resources of the participants as well as due to overstraining the caregivers with AAC-related tasks. The following quote shows the experiences of an AAC consultant with the implementation of the intervention:

*“I think that many have the problem anyway*,* all the theory during the joint care rounds*,* right? The nice formulation of objectives*,* where it seems quite clear what needs to be done*,* but then the transfer into everyday life*,* the practical implementation*,* that’s what often fails. And that’s where the training helps. We can then go into the situation*,* into everyday life and show that’s how it can work and perhaps that’s how it will work. They simply need this*,* watching*,* copying*,* demonstrating*,* the modelling.” (B5AT1M*,* AAC consultant*,* 136–137)*

#### Changes in the workload and work process

In the focus group interviews, the caregivers and the AAC consultants reported that the intervention caused additional workload, which was not sufficiently considered:

*“So I think it’s really nice*, *name5* comes to our school for two hours,* takes the time. But it’s on us to organise everything around it. That takes me away from my daily tasks*,* then colleagues are out sick*,* and that’s already… I really enjoy doing it all*,* but it takes a lot of effort.” (B3BT1B#2*,* teacher*,* 142).*

#### Unintended effects of the intervention

During the focus group interviews, the transfer of AAC knowledge to other stakeholders and the positive effects on other children with AAC needs who did not participate in the intervention were reported. The intervention required many AAC counselling centres’ resources, so that in some cases there was not enough time for case discussions. In addition, some stakeholders were described as less willing to participate in the intervention process:

*“They all sit here in the consultation session and… so they come to consultation first of all with a positive attitude*,* an open mind*,* and usually… nod when you say*,* that’s what needs to be done. That’s how we want to do it. But when it comes to doing it*,* when it becomes clear that it needs to happen in their context*,* because the child is with them most of the time*,* six hours in daycare*,* or eight hours at school or in the sheltered workshop*,* then their willingness to participate is different*,* depending on the situation.” (B2BT1M#1*,* AAC consultant*,* 317)*

### Intermediate outcomes

#### Acceptance, use competence, and use of the AAC system

The survey results showed that for 4 out of 5 items on the acceptance of the caregiver towards the new AAC system (Table 2), the mean values in the intervention group were higher than those in the comparison group at T2. These differences are statistically significant for 3 out of 5 items. The values of the subscale ‘Acceptance of the AAC user towards the new AAC system’ are rather low compared to the other three subscales (these mean scores are all around, or less than 2) and in particular compared to the values of the acceptance of the caregivers towards the new AAC system. The mean scores of the 7 items related to the AAC user’s acceptance of the new AAC system were higher in the intervention group at T2; however, the difference was not statistically significant.

**Table 2:** Acceptance, use competence, and use of the AAC system

Items	Intervention Group		Comparison Group		
	M	SD	M	SD	MWU (p)
Acceptance of the caregiver towards the new AAC system					
1. I am motivated to use the new AAC system with the person.	3.37	0.90	3.15	1.00	0.025*
2. I accept that the person is unlikely to speak and will require this or other AAC systems for the foreseeable future.	3.34	1.06	3.28	1.00	0.382
3. I do not find it frustrating to use the new AAC system with the person.	3.31	1.02	3.07	1.10	0.035*
4. I can realistically assess what impact the new AAC system could have on the person’s quality of life.	3.10	0.95	2.84	1.04	0.024*
5. I believe that the person needs the new AAC system in order to communicate more effectively with me.	3.05	1.20	3.10	1.13	0.886
Subscale sum scores	3.23	0.77	3.09	0.85	0.156
Acceptance of the AAC user towards the new AAC system					
1. The person prefers the new AAC system over other, simpler forms of communication such as vocalisations, gestures, and less complex forms of communication.	1.84	1.25	1.63	1.10	0.172
2. The person had a say in selecting the new AAC system.	1.81	1.44	1.78	1.38	0.878
3. The person appears to value the new AAC system.	2.61	1.24	2.47	1.09	0.213
4. When using the new AAC system, the person does not appear to get frustrated as often as with other forms of communication.	2.25	1.23	2.09	1.16	0.217
5. The person demonstrates a sense of ownership of the AAC device.	2.57	1.48	2.23	1.50	0.062
Use competence of the caregiver and the AAC user					
1. The person is usually more successful communicating with the new AAC system than with other forms of communication.	2.35	1.22	2.24	1.14	0.361
2. The person conveys his/her feelings, wishes and needs more effectively with the new AAC system than with other forms of communication.	2.03	1.18	1.89	1.22	0.328
3. The person is physically able to use the new AAC system. For example, he/she can press the buttons/show symbols/perform gestures.	3.28	0.94	3.15	1.04	0.253
4. The person is able to use the new AAC system independently without needing help.	2.19	1.28	1.85	1.27	0.024*
5. The person understands how to use the new AAC system.	2.77	1.15	2.57	1.02	0.057
6. It is easy to programme messages on the AAC device (e.g., on a voice output device) or to design content for the AAC device (e.g., new symbol cards, me-book content).	2.78	1.13	2.71	1.13	0.550
7. The new AAC system enables the person to communicate for a variety of purposes such as making requests, choices, responding to questions, and disagreeing.	2.53	1.18	2.34	1.18	0.149
Use of the new AAC system in everyday life					
1. I encourage the person to use the new AAC system both at home and in the community.	3.06	1.08	2.77	1.09	0.009*
2. I make sure the new AAC system is available to the person whenever possible.	3.36	0.94	2.82	0.98	< 0.001**
3. There are reasons for the person to use the new AAC system frequently at home and elsewhere.	2.60	1.19	2.47	1.17	0.307
4. The person has numerous opportunities to use the new AAC system.	2.91	1.01	2.54	1.03	0.001*
5. It is easy to use the new AAC system in daily interactions with the person.	2.63	1.04	2.19	0.98	< 0.001**
6. The use of the new AAC system is more appropriate than other forms of communication the person has previously used.	2.65	1.11	2.56	1.07	0.500
7. Communication with the person is usually more effective when he/she uses the new AAC system rather than other forms of communication.	2.37	1.13	2.17	1.10	0.167

*M* mean value, *SD* standard deviation, *MWU* Mann-Whitney U-test

Significant results are marked with *, *p*=significance level: **statistically significant at 1% level, *statistically significant at 5% level

In the focus group interviews, a high level of acceptance of the AAC system among the AAC users and their caregivers was reported. AAC users were described as curious and interested in the AAC system. In contrast, adult AAC users were reported to sometimes find it more difficult to show interest in the AAC system, as old behavioural patterns and habits had to be changed. Many caregivers showed high acceptance of the AAC system and were highly committed. Problems in acceptance by some formal caregivers were also reported, and these problems had a strong negative impact on the success of AAC implementation. The predominantly high acceptance of the AAC system by the AAC users is made evident in the following quote from a teacher:

*“Yes*,* and to see the progress that is being made and also*,* what I find particularly great in this case*,* their own acceptance*,* the need*,* I’m using this now and I’m coming to… am also recognised by my environment*,* that’s just great*,* the process that was possible.” (B4AT1B#2*,* teacher*,* 253)*

In terms of the use competence the quantitative data indicate that all items related to the use competence of the AAC system had higher mean values in the intervention group than in the comparison group; however, the difference was only statistically significant for one item ‘independent use of the AAC system’ (Table 2). For all 7 items related to the use of the new AAC system in everyday life, 4 items showed a statistically significant difference in favour of the intervention group.

In the focus group interviews the competence of AAC users and caregivers in using the AAC system was described positively. In this context, it was emphasised that AAC training and therapy were very helpful:

*“Because*,* as I said*,* I think it would not have worked out for them*,* if they had been handed this AAC system without guidance.” (CT1M*,* AAC consultant*,* 175)*

The focus group participants reported that it was more difficult for some caregivers to learn how to use the AAC system, as it took much time and effort to learn about the use of the AAC system (e.g., location of categories).

#### Intervention compliance

Qualitative data suggested that compliance with the use of the AAC system in everyday life depended strongly on the motivation of the informal caregivers. As the effort required to use the AAC system in different contexts was relatively high, the motivation of the informal caregivers was perceived as not always sufficient:

*“I believe*,* however*,* that the compliance of the caregivers affects the compliance of the AAC users significantly… because communication has so much to do with relationships.” (BT1M*,* AAC consultant*,* 164)*

### Context factors

#### Previous experience with AAC

The quantitative data show that the self-assessed previous experience of the caregivers did not differ significantly between the intervention and comparison groups (Table 3). The items used are scaled from 0 to 4 (“no experience at all” - “very much experience”). Previous experience with the use of AAC systems was described in the focus group interviews as beneficial and thus represents a relevant contextual factor.

**Table 3:** Descriptive results on the previous experience with AAC

	Intervention group (*n* = 127)		Comparison goup (*n* = 91)		MWU (p)
	M	SD	M	SD	
Previous experience with electronic AAC systems	1.13	1.19	1.30	1.30	0.386
Previous experience with non-electronic AAC systems	1.54	1.19	1.56	1.27	0.966
Previous experience with gestures	1.24	1.06	1.34	1.22	0.745

*M* mean value, *SD* standard deviation, *MWU* Mann-Whitney U-test

#### Stakeholders’ attitudes towards AAC

The attitudes of caregivers were described in the focus group interviews as a facilitating or inhibiting contextual factor influencing AAC implementation. These included, for example, concerns about the effort associated with AAC use, too high expectations and openness towards AAC, as well as the motivation to try something new. These attitudes often hindered the use of AAC:

*“Yes*,* the child can definitely communicate better. As I said*,* unfortunately it still can’t use sign language very well. It also won’t be practised at home. We tried to implement it in daycare*,* but of course we reach our limits with that too.” (B1AT1B#1*,* educator*,* 124)*

It was also reported that caregivers can influence each other, as the following quote illustrates:

*“Now*,* for example*,* the family I support*, the mother wasn’t open to it at first. I had printed out pictures for her in *aids_symbol cards*, *name2*,* and she didn’t use them at first for fear that they would get wet on the way home. […] And it took me a long time to convince her to accept it at all. And that is now… she’s becoming more open now.” (B3AT1B#1*,* Special needs teacher*,* 250–252)*

#### Technology commitment

Concerning the quantitative data neither caregivers in the intervention nor in the comparison group showed particularly high or low technology commitment values (intervention group: mean = 3.64; comparison group: mean = 3.77). The topic of technology commitment was not raised in the focus group interviews, which underlines the low relevance of this context factor.

#### Framework conditions

The results of the focus group interviews showed that existing regulations on responsibilities and the determination of AAC needs are perceived as inadequate and, in some cases, not conducive to the objectives of the intervention. The following quote demonstrates the potential difficulties in determining AAC needs:

*“So I think that the ongoing issue is still… […] that some health insurance companies simply don’t approve the AAC consultation and that has always been a bit of an issue for us. […] And that*,* I think*,* will… we have to continue to pursue the issue that somehow a consensus can be reached and that everyone who has a need actually gets the AAC consultation payed for. […] And the second point that I also see*,* which is also an ongoing issue*,* is that the health insurance companies handle the provision of AAC systems very differently*,* I would say. Some don’t check at all and wave it through. And some check down to the last detail. […]” (B2BT1M#1*,* AAC consultant*,* 220)*

As the quote shows, the financial framework conditions with regard to the application process and the funding of services and AAC systems were also described as insufficient and partly arbitrary. In addition, the time resources of all participants were a critical factor for the implementation of the intervention. The coordination of appointments with limited time resources for all participants was a great challenge for the AAC consultants.

#### Stakeholders’ collaboration and communication

The focus group participants reported that the collaboration and communication between all stakeholders in the AAC user network were important factors for the success of AAC care:

*“And that shows once again how important it is that the whole team is able to do it. And yes*,* they are very motivated. And they want more now. Learn more*,* experience more.” (B3AT1B#1*,* special needs teacher*,* 72)*.

#### Caregiver burden

The often high informal caregiver burden was reported to influence the extent to which the intervention was implemented in everyday life:

*“Even if it sometimes seems that perhaps the parents are not into it*,* perhaps they would like to participate*,* but they just don’t know how to do it or find the time for it. Because we’ve had a second child in September. And when everyone is finally asleep in the evening*,* we are exhausted and then it’s sometimes difficult to focus and get back to dealing with the AAC system.” (B2AT1B#1*,* father*,* 291–293)*

#### Characteristics and personal background of AAC users

The AAC users in the intervention group are very heterogeneous, e.g., in terms of age, type of disability or motor and cognitive impairments (see Supplement). In the focus group interviews, the participants reported that it was more difficult or easier for the AAC users to implement the AAC system, depending on their personal characteristics:

*“And in the meantime we also thought about using electronic devices because we have a problem with my son’s motor skills. He has muscular hypotonia*,* which means that sign language is out of the question at the moment. And we started with two little symbol cards*,* with eating and drinking. And then he was supposed to point to them with his little hand and that didn’t really work… He always pointed to one card*,* even when we changed them. And then we didn’t know if it worked for him at all because he is also cognitively impaired.” (B6AT1B#2*,* mother*,* 30)*

### Needs for adaptation

The needs for adaptation of the intervention identified by the focus group participants included the following:

- AAC care should be implemented at an earlier age
- Better and more intensive collaboration between all stakeholders
- Supervision of AAC consultants and professional exchange
- Further development of case management structures and a suitable documentation management
- Reimbursement of travel time and costs of AAC consultants (for AAC therapy appointments)
- More transparency in the AAC system approval process of health insurance companies
- More flexible adaptation of interventions to individual needs and circumstances due to the high heterogeneity of AAC users and extent of support received
- More awareness of and knowledge about AAC among all stakeholders involved, especially physicians and the integration of AAC in the training of speech and language pathologists and special educators
- Continuation of case management after the end of AAC therapy for a sustainable AAC network

## Discussion

In this study, we evaluated the processes of implementing a complex intervention to improve AAC care. On the basis of the MRC process evaluation guidance by Moore et al. [26], qualitative and quantitative data have been integrated to gain a deeper understanding of the functioning and context of the intervention. The results show that the implementation of the intervention varied, e.g., due to longer therapy duration and staff changes. Thus, the implementation of the intervention depended on the characteristics of the AAC users and their previous experience with AAC and the attitudes of their formal and informal caregivers towards AAC. The intervention was difficult to standardise due to the complexity of the intervention, the high heterogeneity within the group of AAC users and caregivers and a variety of context factors, such as the lack of regulation and standardisation of AAC care in the health and social care system. Intervention elements such as AAC assessment and consultation, AAC training, AAC therapy, and case management were highly positively evaluated. AAC training was evaluated as particularly valuable in terms of AAC users’ and their caregivers’ competence in operating and using AAC systems. AAC therapy was also described as particularly helpful in transferring the use of the AAC system into everyday life and into the environment in which the AAC user communicates. Case management was evaluated by stakeholders as essential for managing the often challenging application process within the health insurance system, as well as ensuring sustainable AAC care. The participants in the intervention group felt significantly better supported than the participants in the comparison group, e.g., with regard to setting and pursuing AAC care goals. According to the surveys and focus group interviews, the intervention achieved a high level of acceptance among the participants, and the use competence reportedly improved as a result of the intervention. In terms of the intermediate outcomes investigated (acceptance, use competence, and use of the AAC system), in some domains - as acceptance of the caregiver towards the new AAC system and use of the new AAC system in everyday life - the results suggest a positive influence of the intervention. The literature highlights that holistic integration of informal caregivers is important for increasing the acceptance of AAC [39, 44]. The evaluation confirmed that in the intervention studied, the informal caregivers of AAC users were integrated throughout the entire care process, and this fact was highly valued by all the stakeholders involved.

In addition, it was found that a variety of contextual factors at both the individual and system levels influence AAC care, often independently of the intervention but also as determinants of implementation success. In particular, stakeholders’ attitudes towards AAC seemed to substantially impact intervention implementation, which was also illustrated in an interview study by Moorcroft et al. [45] with parents who rejected AAC. The study showed that stakeholders, e.g., speech and language pathologists, can indeed influence parents’ attitudes towards AAC. Another contextual factor that is often mentioned in the literature as a barrier to the implementation of AAC is a lack of time for formal caregivers (e.g., in day care, school) [8, 46]. For these reasons, implementing complex AAC interventions such as described in our study requires a detailed analysis of the AAC care situation and adequate intervention design approaches that consider the complexity of AAC users’ and caregivers’ characteristics as well as the complexity of the health and social care system.

### Strengths and limitations

The recruitment period had to be extended to reach the required sample size of AAC users enrolled in the intervention for several reasons that may also have had an effect on the implementation of the intervention: (1) the COVID-19 pandemic restricted the activities of the AAC counselling centres and the availability and capacities of AAC users and their caregivers (2), applications for AAC systems were frequently rejected by health insurance companies and thus cases dropped out, and (3) applications for AAC diagnostics and consultation by AAC counselling centres were frequently rejected by health insurance funds in the comparison group.

As in many studies on complex interventions in healthcare, we aimed to reflect the heterogeneity of the group of AAC users. The intervention as well as the evaluation design had to be adapted to the healthcare regulations. There was strong heterogeneity in the characteristics of both AAC users and caregivers. This can be seen as a strength, as it reflects reality and acknowledges that communication impairments can have a variety of backgrounds. While this strengthens the external validity, it also weakens the internal validity. The implementation of the intervention was also rather heterogeneous. Another limitation is that not all aspects of the research questions were addressed equally by both methods, as some elements were explored only qualitatively or quantitatively. This may result in gaps where certain details or generalisable patterns remain underrepresented. The results of the quantitative and qualitative surveys differ in certain categories, with the qualitative findings often being more positive than the quantitative ones. This discrepancy can be partly explained by the fact that the quantitative survey included more specific and detailed questions about individual situations and specific skills. In comparison, the questions in the focus group interviews were more generic. It can also be assumed that focus group participants expressed less criticism in the interview situation, as they possibly wanted to please and/or felt gratitude for the intervention participation and for these reasons did not raise negative comments. In addition, the predefined time periods for the intervention delivery were generally too short. As a result, the data collection periods of the evaluation had in some cases to be individually adapted, which could have led to distortions in the evaluation. Necessary adjustments in the implementation of the intervention during the COVID-19 pandemic, especially the partial switch to teletherapy, also contributed to the heterogeneity of the intervention delivery. The communication and health limitations of AAC users necessitated a proxy survey of informal and formal caregivers on some aspects. However, we conducted individual interviews with AAC users. The results are published elsewhere [47]. Furthermore, some of the formal caregivers changed in the course of the study e.g., due to transitions of the AAC users from day care to school. Although this was unavoidable, it could limit the reliability of the results. The collection of both informal and formal caregiver perspectives can be seen as beneficial for obtaining complementary results. The lack of consideration of the perspective of passive stakeholders (e.g., physicians and health insurance companies) can be seen as a limitation.

## Conclusion

The process evaluation in a mixed methods design allowed valuable deeper insights into AAC care in Germany and into the complexity of an intervention to improve AAC care. The intervention was rated predominantly positively by caregivers and AAC consultants, with intensive therapy sessions supporting the integration of AAC into daily life and case management helping to address challenges and foster collaboration. This evaluation reflects the needs and challenges of the participants and identifies facilitators and barriers to implementation that can inform adaptations in future interventions. The discussed methodological limitations point to the high relevance of pilot studies and methodological work in this field, which could significantly promote the internal validity of such intervention studies. The identified needs for adaptation (e.g., supervision of AAC consultants, continuation of case management) as well as the presented relevant contextual factors may help to adapt future interventions. The following aspects should be taken into account when developing comparable future interventions: longer intervention periods (longer than 6 months), flexible adaptation of the therapy setting to the individual situation and seamless transition to other therapy formats (e.g., speech therapy with an AAC focus) after the end of the intervention. With regard to future studies, it might be advisable to limit heterogeneity and to stratify for health conditions and age groups. Beyond the evaluation results, this study was able to reveal numerous findings about AAC care in Germany, the challenges and burdens of the stakeholders involved, their support needs, and the relevance of functioning collaboration among the stakeholders [6, 7, 20, 39].

## Supplementary Information

Supplementary Material 1.

Supplementary Material 2.

Supplementary Material 3.

## Data Availability

The datasets used and/or analysed during the current study are available from the corresponding author on reasonable request.

## Abbreviations

AAC: Augmentative and alternative communication
SGB: Soziales Gesetzbuch [Social Security Code]
MRC: Medical Research Council
M: Mean
SD: Standard deviation
MWU: Mann-Whitney U-test
