# Supplementary material for: Process evaluation of a complex intervention in augmentative and alternative communication care in Germany: a mixed methods study
Source: BMC Health Serv Res. 2025 Mar 13;25:373. doi: 10.1186/s12913-025-12452-y (PMC11905436; doi:10.1186/s12913-025-12452-y)
Supplement: Supplementary file 1 — Supplementary Material 1. [file 12913_2025_12452_MOESM1_ESM.docx]

**Interview guideline for focus group interviews with AAC consultants**

| **Topic** | **Main questions** | **Further questions** | **Indicators** |
| --- | --- | --- | --- |
| **Implementation of the intervention** | - **How was the intervention practiced?** - **What are your experiences with the intervention?** - What kind of practical preparation was needed to implement the project? | - How is case management implemented? And how do you rate this implementation? - What is the difference between AAC training and AAC therapy? - Questions about the essential elements of the intervention:   - AAC consultation   - AAC training   - AAC therapy   - case management | Preconditions, input  Structures, processes  Implementation  Assessment of feasibility |
| **Changes since the implementation of the intervention** | - **How would you rate the effects of the intervention?** - Has anything changed for you since the intervention was implemented? - How do you assess the benefits of the intervention, especially with regard to the project's goals of improving communication skills, quality of life, satisfaction with the AAC system and the participation of AAC users?   We now have a more interactive task for you on this topic.  Last year, before the start of the project, we also conducted focus group interviews in which we asked, among other things, what the wishes for ideal AAC care are. As you can see, we have now written these wishes on cards. We will now go through each wish with you and ask you to decide whether the aspect has worsened, remained unchanged or has improved as a result of the intervention. | - Changes in work processes or organisation? - Changes in the workload? (e.g., working time, travelling time) - Changes in resource use? (e.g., material resources, consultation materials) - Reaction of the participants? (use competence, acceptance, compliance) - In your opinion, which intervention measures (e.g., AAC consultation, AAC training, AAC therapy and case management) have the greatest benefit for the AAC users (e.g., to improve communication or quality of life)? | Changes since status quo  Achievement of the intended goal  Changes with regard to use competence, acceptance, and compliance  Changes in outcomes |
| **Need for adaptation** | - **What adaptations need to be made to the intervention?** - Do you have any suggestions for improving or adapting the intervention? | - To what extent may AAC consultation/AAC training/AAC therapy/case management need to be adapted? - Is there a need to adapt the intervention to meet the needs of the AAC user? - Is there a need for adaptation of financing? | Need for adaptation |
