# Supplementary material for: Process evaluation of a complex intervention in augmentative and alternative communication care in Germany: a mixed methods study
Source: BMC Health Serv Res. 2025 Mar 13;25:373. doi: 10.1186/s12913-025-12452-y (PMC11905436; doi:10.1186/s12913-025-12452-y)
Supplement: Supplementary file 2 — Supplementary Material 2. [file 12913_2025_12452_MOESM2_ESM.docx]

**Interview guideline for focus group interviews with informal and formal caregivers**

| **Topic** | **Main questions** | **Further questions** | **Indicators** |
| --- | --- | --- | --- |
| **Implementation of the intervention** | - **How was the intervention practiced?** - How has the care process been for you since your first contact with the counselling centre? - **What are your experiences with the intervention?** - How is the implementation of the intervention compatible with your everyday work or private life? | - How was/is the AAC consultation, AAC training and AAC therapy - depending on where you are in the care process? - Have there been or are there any problems in your care (e.g., in applying for AAC systems) for which you receive or have received support from the counselling centre? - **Formal caregivers:** Implementation in work contexts such as day care, schools, sheltered workshops, and homes for persons with disabilities - **Informal caregivers**: Implementation, for example, in everyday life, at home, in day care or at school | Preconditions, input  Structures, processes  Implementation  Assessment of feasibility |
| **Changes since the implementation of the intervention** | - **How would you rate the effects of the intervention?** - Has anything changed since the start of the project? - How do you assess the benefits of the intervention, especially with regard to the project's goals of improving communication skills, quality of life, satisfaction with the AAC system and the participation of AAC users?   We now have a small, more interactive task for you on this topic.  Last year, before the start of the project, we also conducted focus group interviews in which we asked, among other things, what the wishes for ideal AAC care are. As you can see, we have now written these wishes on cards. We will now go through each wish with you and ask you to decide whether the aspect has worsened, remained unchanged or has improved as a result of the intervention. | - - Has the intervention (e.g., AAC training) changed the way you use the AAC system in your everyday life? - In your opinion, which intervention measures (e.g., AAC consultation, AAC training, AAC therapy and case management) have the greatest benefit for the AAC user (e.g., to improve communication skills or quality of life)? | Changes since status quo  Achievement of the intended goal  Changes with regard to use competence, acceptance, and compliance  Changes in outcomes |
| **Need for adaptation** | - **What adaptations need to be made to the intervention?** - Do you have any suggestions for improving or adapting the intervention? | - Did you receive sufficient support from the counselling centre? - Is there a need to adapt the intervention to meet the needs of the AAC user? - To what extent may AAC consultation/AAC training/AAC therapy/case management need to be adapted? - Is there a need for adaptation of financing? | Need for adaption |
