# Supplementary material for: Process evaluation of a complex intervention in augmentative and alternative communication care in Germany: a mixed methods study
Source: BMC Health Serv Res. 2025 Mar 13;25:373. doi: 10.1186/s12913-025-12452-y (PMC11905436; doi:10.1186/s12913-025-12452-y)
Supplement: Supplementary file 3 — Supplementary Material 3. [file 12913_2025_12452_MOESM3_ESM.docx]

**Supplement: Sample description of AAC users and caregivers**

| Sample description of AAC users | | | | | | | | |
| --- | --- | --- | --- | --- | --- | --- | --- | --- |
|  | All cases  (T0) | | | | Valid cases  (T0 + T2 are present) | | | |
|  | **Intervention group** | | **Comparison group** | | **Intervention group** | | **Comparison group** | |
|  | n (%) | | n (%) | | n (%) | | n (%) | |
| Total | 152 (100) | | 104 (100) | | 127 (100) | | 91 (100) | |
| Age (in years) |  | |  | |  | |  | |
| 0–2 | 1 (0.7) | | 4 (3.8) | | 1 (0.8) | | 4 (4.4) | |
| 3–4 | 23 (15.1) | | 18 (17.3) | | 22 (17.3) | | 14 (15.4) | |
| 5–6 | 31 (20.4) | | 23 (22.1) | | 29 (22.8) | | 21 (23.1) | |
| 7–10 | 31 (20.4) | | 20 (19.2) | | 24 (18.9) | | 19 (20.9) | |
| 11–14 | 23 (15.1) | | 17 (16.3) | | 15 (11.8) | | 14 (15.4) | |
| 15–19 | 8 (5.3) | | 6 (5.8) | | 8 (6.3) | | 4 (4.4) | |
| 20–29 | 12 (7.9) | | 5 (4.8) | | 9 (7.1) | | 4 (4.4) | |
| 30–39 | 7 (4.6) | | 4 (3.8) | | 7 (5.5) | | 4 (4.4) | |
| 40–49 | 3 (2) | | 4 (3.8) | | 3 (2.4) | | 4 (4.4) | |
| 50–59 | 8 (5.3) | | 1 (1) | | 6 (4.7) | | 1 (1.1) | |
| 60–69 | 4 (2.6) | | 1 (1) | | 2 (1.6) | | 1 (1.1) | |
| über 69 | 1 (0.7) | | 1 (1) | | 1 (0.8) | | 1 (1.1) | |
| Sex |  | |  | |  | |  | |
| Female | 54 (35.5) | | 31 (29.8) | | 45 (35.4) | | 27 (29.7) | |
| Male | 98 (64.5) | | 73 (70.2) | | 82 (64.6) | | 64 (70.3) | |
| Cause of disability |  | |  | |  | |  | |
| Congenital | 132 (86.8) | | 88 (84.6) | | 114 (89.8) | | 78 (85.7) | |
| Accident | 1 (0.7) | | 4 (3.8) | | 1 (0.8) | | 3 (3.3) | |
| Disease | 12 (7.9) | | 12 (11.5) | | 7 (5.5) | | 10 (11) | |
| Others | 7 (4.6) | | 0 (0) | | 5 (3.9) | | 0 (0) | |
| Disability |  | |  | |  | |  | |
| Specific genetic syndromes | 22 (14.5) | | 20 (19.2) | | 22 (17.3) | | 18 (19.8) | |
| Down-Syndrom | 21 (13.8) | | 10 (9.6) | | 20 (15.7) | | 8 (8.8) | |
| Unspecified developmental disorders | 28 (18.4) | | 19 (18.3) | | 21 (16.5) | | 17 (18.7) | |
| Autism spectrum disorder | 41 (27) | | 23 (22.1) | | 37 (29.1) | | 21 (23.1) | |
| Cerebral movement disorders | 20 (13.2) | | 18 (17.3) | | 14 (11) | | 16 (17.6) | |
| Neuromuscular diseases | 1 (0.7) | | 1 (1) | | 1 (0.8) | | 1 (1.1) | |
| Stroke / brain haemorrhages | 3 (2) | | 0 (0) | | 2 (1.6) | | 0 (0) | |
| Traumatic brain injury | 2 (1.3) | | 3 (2.9) | | 1 (0.8) | | 2 (2.2) | |
| (Early childhood) acquired brain damage due to various causes | 7 (4.6) | | 4 (3.8) | | 3 (2.4) | | 3 (3.3) | |
| Various congenital brain malformations | 5 (3.3) | | 1 (1) | | 4 (3.1) | | 1 (1.1) | |
| Missing diagnosis | 2 (1.3) | | 5 (4.8) | | 2 (1.6) | | 4 (4.4) | |
| Employment situation |  | |  | |  | |  | |
| Is cared for exclusively at home | 12 (7.9) | | 5 (4.8) | | 9 (7.1) | | 4 (4.4) | |
| Attends the crèche / day care / kindergarten | 52 (34.2) | | 42 (40.4) | | 48 (37.8) | | 36 (39.6) | |
| Attends school | 61 (40.1) | | 41 (39.4) | | 46 (36.2) | | 36 (39.6) | |
| In vocational training (apprenticeship) | 0 (0) | | 0 (0) | | 0 (0) | | 0 (0) | |
| Attends a higher education institution (e.g., technical college, university) | 0 (0) | | 0 (0) | | 0 (0) | | 0 (0) | |
| Is employed (regular labour market) | 0 (0) | | 1 (1) | | 0 (0) | | 1 (1.1) | |
| Is employed in a social institution (e.g., sheltered workshop) | 22 (14.5) | | 14 (13.5) | | 20 (15.7) | | 13 (14.3) | |
| Others | 5 (3.3) | | 1 (1) | | 4 (3.1) | | 1 (1.1) | |
| Living situation |  | |  | |  | |  | |
| Together with parents or other guardians | 112 (73.7) | | 91 (87.5) | | 97 (76.4) | | 78 (85.7) | |
| Together with the (spousal) partner | 4 (2.6) | | 1 (1) | | 3 (2.4) | | 1 (1.1) | |
| in a social institution | 34 (22.4) | | 12 (11.5) | | 26 (20.5) | | 12 (13.2) | |
| alone (with assistance if necessary) | 1 (0.7) | | 0 (0) | | 0 (0) | | 0 (0) | |
| Others | 1 (0.7) | | 0 (0) | | 1 (0.8) | | 0 (0) | |
| Sample description of informal and formal caregivers | | | | | | | | |
|  | | **All cases**  **(T0)** | | | | **Valid cases**  **(T0 + T2 are present)** | | |
|  | | **Intervention group** | | **Comparison group** | | **Intervention group** | | **Comparison group** |
|  | | n (%) | | n (%) | | n (%) | | n (%) |
| Total | | 255 (100) | | 146 (100) | | 193 (100) | | 123 (100) |
| Age (in Years) | |  | |  | |  | |  |
| 0–29 | | 40 (15.7) | | 17 (11.6) | | 33 (17.1) | | 14 (11.4) |
| 30–39 | | 89 (34.9) | | 37 (25.2) | | 68 (35.2) | | 30 (24.4) |
| 40–49 | | 77 (30.2) | | 57 (39) | | 61 (31.6) | | 46 (37.4) |
| 50–59 | | 40 (15.7) | | 29 (19.9) | | 24 (12.4) | | 29 (23.6) |
| 60–69 | | 9 (3.5) | | 6 (4.1) | | 7 (3.6) | | 4 (3.3) |
| Sex | |  | |  | |  | |  |
| Female | | 205 (80.4) | | 124 (84.9) | | 155 (80.3) | | 104 (84.6) |
| Male | | 50 (19.6) | | 22 (15.1) | | 38 (19.7) | | 19 (15.4) |
| Caregivers | |  | |  | |  | |  |
| informal | | 122 (47.8) | | 61 (41.8) | | 87 (45.1) | | 51 (41.5) |
| formal | | 133 (52.2) | | 85 (58.2) | | 106 (54.9) | | 72 (58.5) |
| Formal caregivers: Occupational group | |  | |  | |  | |  |
| Teacher | | 39 (32) | | 19 (31.1) | | 24 (27.6) | | 15 (29.4) |
| Educator (e.g., in day care) | | 22 (18) | | 6 (9.8) | | 18 (20.7) | | 4 (7.8) |
| Therapist (e.g., speech and language pathologist) | | 32 (26.2) | | 27 (44.3) | | 24 (27.6) | | 23 (45.1) |
| Nurse or personal assistant | | 2 (1.6) | | 0 (0) | | 2 (2.3) | | 0 (0) |
| Employee in residential or social facilities (e. g., homes for persons with disabilities, sheltered workshop) | | 21 (17.2) | | 6 (9.8) | | 17 (19.5) | | 6 (11.8) |
| Others | | 6 (4.9) | | 3 (4.9) | | 2 (2.3) | | 3 (5.9) |
| Informal caregivers: relationship to the AAC user | |  | |  | |  | |  |
| (Spousal) partner | | 3 (2.3) | | 1 (1.2) | | 2 (1.9) | | 1 (1.4) |
| Mother/father | | 100 (75.2) | | 76 (89.4) | | 84 (79.2) | | 64 (88.9) |
| Other legal guardian | | 1 (0.8) | | 0 (0) | | 1 (0.9) | | 0 (0) |
| Relative | | 1 (0.8) | | 2 (2.4) | | 1 (0.9) | | 1 (1.4) |
| Girlfriend | | 1 (0.8) | | 0 (0) | | 0 (0) | | 0 (0) |
| Other | | 27 (20.3) | | 6 (7.1) | | 18 (17) | | 6 (8.3) |
| Education | |  | |  | |  | |  |
| Left school without qualifications | | 6 (2.4) | | 2 (1.4) | | 5 (2.6) | | 2 (1.6) |
| German primary school qualification | | 2 (0.8) | | 0 (0) | | 2 (1) | | 0 (0) |
| German basic school qualification | | 12 (4.7) | | 10 (6.8) | | 8 (4.1) | | 8 (6.5) |
| German secondary school certificate | | 49 (19.2) | | 20 (13.7) | | 39 (20.2) | | 16 (13) |
| German advanced technical college qualification | | 85 (33.3) | | 42 (28.8) | | 70 (36.3) | | 36 (29.3) |
| German higher school certification | | 101 (39.6) | | 72 (49.3) | | 69 (35.8) | | 61 (49.6) |
